# Shrimp miR-10a Is Co-opted by White Spot Syndrome Virus to Increase Viral Gene Expression and Viral Replication

**DOI:** 10.3389/fimmu.2017.01084

**Published:** 2017-09-06

**Authors:** Jiun-Yan Huang, Shih-Ting Kang, I-Tung Chen, Li-Kwan Chang, Shih-Shun Lin, Guang-Hsiung Kou, Chia-Ying Chu, Chu-Fang Lo

**Affiliations:** ^1^Department of Biotechnology and Bioindustry Sciences, College of Bioscience and Biotechnology, National Cheng Kung University, Tainan, Taiwan; ^2^Center for Shrimp Disease Control and Genetic Improvement, National Cheng Kung University, Tainan, Taiwan; ^3^Department of Biochemical Science and Technology, College of Life Science, National Taiwan University, Taipei, Taiwan; ^4^Department of Life Science, National Taiwan University, Taipei, Taiwan; ^5^Institute of Biotechnology, National Taiwan University, Taipei, Taiwan; ^6^Center for Systems Biology, National Taiwan University, Taipei, Taiwan

**Keywords:** white spot syndrome virus, microRNA, miR-10a, virus–host interaction, *Penaeus vannamei*

## Abstract

Members of the microRNA miR-10 family are highly conserved and play many important roles in diverse biological mechanisms, including immune-related responses and cancer-related processes in certain types of cancer. In this study, we found the most highly upregulated shrimp microRNA from *Penaeus vannamei* during white spot syndrome virus (WSSV) infection was miR-10a. After confirming the expression level of miR-10a by northern blot and quantitative RT-PCR, an *in vivo* experiment showed that the viral copy number was decreased in miR-10a-inhibited shrimp. We found that miR-10a targeted the 5′ untranslated region (UTR) of at least three viral genes (*vp26, vp28*, and *wssv102*), and plasmids that were controlled by the 5′ UTR of these genes produced enhanced luciferase signals in transfected SF9 cells. These results suggest a previously unreported role for shrimp miR-10a and even a new type of host–virus interaction, whereby a co-opts the key cellular regulator miR-10a to globally enhance the translation of viral proteins.

## Introduction

MicroRNAs (miRNAs) are short, non-coding, endogenous RNAs with a length of 17–25 nucleotides (nt). They are involved in posttranscriptional regulation, which they usually achieve by targeting the untranslated regions (UTR) of an mRNA (1–3). miRNAs are key regulators and play an important role in diverse biological systems, including cell proliferation, cell differentiation, metabolism, development, apoptosis, and host–pathogen interactions (4, 5). Recent studies have demonstrated that viral infection can alter the expression of cellular miRNA species in the cells of the host, and large changes in the expression of cellular miRNAs can impact virus replication either directly by targeting viral nucleic acid sequences (6–10) or else indirectly by targeting cellular mRNAs (11, 12).

Shrimp white spot syndrome virus (WSSV) encodes more than a 100 viral miRNAs, and most of these are expressed from the early stage of viral infection (13). WSSV-miR-66 and WSSV-miR-68 promote WSSV infection by targeting viral genes (14), while WSSV-miR-N13 and WSSV-miR-N23 help the virus by targeting the host antiviral Dorsal gene (15). WSSV-miR-N24 targets the shrimp caspase 8 gene and represses the shrimp apoptosis activity (16). Meanwhile, although WSSV uses the host defense gene STAT to promote the expression of IE1 (17), these is also evidence that WSSV-miR-22 benefits the virus by targeting and downregulating the host STAT gene (18).

Recent studies have shown not only that WSSV-encoded miRNAs are involved in host–pathogen interactions but also that the shrimp miRNA expression profile is altered in response to WSSV infection (19). In the lymphoid organs of *Marsupenaeus japonicas*, 63 host miRNAs were differentially expressed after WSSV infection (20). The same research group also found that shrimp miR-7 was upregulated after WSSV infection and that it targeted the 3′ UTR of the WSSV early gene *wsv477* to inhibit viral replication (21). They subsequently found that shrimp miR-100 regulates the apoptosis activity against WSSV infection (22), and it has very recently been reported that miR-100 is involved in shrimp immunity against WSSV infection (23). Other recent findings include shrimp miR-965, which targets *wsv240* and decreases viral replication (24), and miR-9041 and miR-9850, which regulate and decrease STAT expression (25). Thus as might be expected, in all of the above reports, shrimp miRNAs defend against WSSV infection, which viral miRNAs promote viral infection.

In the present study, after using next-generation sequencing (NGS) technology to identify differentially expressed shrimp miRNAs in the stomachs of WSSV-infected *Penaeus vannamei*, we focus on miR-10a, which was the most highly expressed host miRNA after WSSV infection. miR-10a is a member of the highly conserved miR-10 family (26), and unexpectedly, instead of inhibiting viral replication by targeting the 3′ UTR of viral genes, this host miRNA in fact promotes viral replication by directly targeting the 5′ UTR of at least three viral genes (*vp26, vp28*, and *wssv102*) to enhance their translation. This study, therefore, presents a mechanism not previously reported in an invertebrate virus whereby its pathogenesis is enhanced by co-opting a host miRNA.

## Materials and Methods

### Experimental Animals

The Pacific white shrimp *P. vannamei* used in this study were all WSSV-negative, as confirmed by using an IQ2000™ WSSV diagnostic kit (GeneReach Biotechnology Corp.). The shrimp (mean weight 4 g) were purchased from the Aquatic Animal Center in National Taiwan Ocean University and were acclimatized in the laboratory in water tanks with a salinity of 30 ± 1 ppt at 26 ± 1°C for at least 3–5 days before the experiments.

### Preparation of WSSV Inoculum

The virus used in this study was the WSSV Taiwan isolate WSSV-1-Tw (GenBank accession no. AF440570) (27), which was purified from WSSV-infected *Penaeus monodon* shrimp collected in Taiwan in 1994 (28). The WSSV inoculum was prepared as described previously (29). Briefly, a 0.5 g frozen sample of infected *P. monodon* carapace was ground with 4.5 ml of 0.9% NaCl. The homogenized mixture was centrifuged at 1,000 × *g* for 10 min at 4°C, and the supernatant was then filtered with a 0.45-µm filter (Millipore). The resulting filtrate was diluted 100× with phosphate-buffered saline (PBS), and 100 µl of the viral suspension was injected into adult, specific-pathogen-free *P. vannamei* (mean weight 35 g) to induce WSSV infection. Collected hemolymph from moribund shrimp was then centrifuged at 1,000 × *g* for 10 min at 4°C, and the supernatant was diluted 5× with cold PBS and portioned out. The suspension was stored at −80°C and used as a viral stock.

### RNA Extraction

Total RNA was isolated from the stomachs or pleopods of WSSV-negative (0 h-postinfection, hpi) or WSSV-infected shrimp (12, 24, 36, and 48 hpi) using TRIzol reagent (Invitrogen) as described in the manufacturer’s instructions. Briefly, 1 ml of TRIzol reagent was added to each 100 mg of shrimp tissue, and the tissue was homogenized in liquid nitrogen with a mortar and pestle. Chloroform was added (200 µl per 1 ml of TRIzol reagent), and after vigorous shaking, the mixture was allowed to incubate at room temperature for 2–3 min. After centrifuging the sample at 12,000 × *g* for 15 min at 4°C, RNA was precipitated by adding 0.5 ml of isopropanol to the aqueous phase. The RNA was pelleted by centrifugation at 12,000 × *g* at 4°C for 15 min and then washed with 1 ml of 80% ethanol. The RNA pellet was air-dried for 5–10 min, re-suspended in nuclease-free water, and stored at −80°C until use.

### Measuring the Percentage of Small RNA in the Total RNA Samples

The RNA concentration in the shrimp stomach samples was quantitated by using a NanoDrop Spectrophotometer, and the samples were adjusted by dilution to a final RNA concentration of 50–100 ng/µl. The total RNA concentration in a 1-µl sample was then measured using an Agilent^®^ 2100 Bioanalyzer (Agilent) with an RNA 6000 Nano Chip and 2100 Expert Software according to the instructions of the respective manufacturers. Next, the same system was used with a Small RNA Chip to determine the amount of small RNA (defined as 10–40 nts) in another 1 µl sample. The relative small RNA content in each RNA sample was then calculated using the following formula:

% small RNA=(mass of small RNA[10−40nt]from the Small RNA Chip/mass of total RNA from the RNA6000Nano Chip)×100.

The percentage of small RNA content in our RNA samples was always more than 5%, which is 10× greater than the threshold value of 0.5%, below which an enrichment step would be required.

### Construction of the Amplified miRNA Library

MicroRNA libraries used for SOLiD4 sequencing were prepared according to the manufacturer’s instruction (Lifetechnologies). Briefly, 1 µg of total RNA (3 µl) was mixed with 3 µl of Hybridization Solution and 2 µl of SOLiD™ Adaptor Mix in a 0.5-ml PCR tube on ice, and after slowly pipetting up and down a few times, the mixture was briefly spun. After heating to 65°C for 10 min and cooling to 16°C for 5 min, RNA ligation reagents (8 µl of 2× Ligation Buffer and 2 µl of Ligation Enzyme Mix) were added to the hybridization mixture. The mixture was again slowly pipetted up and down a few times to mix well and spun briefly. The 20 µl ligation reaction was then maintained at 16°C for 16 h in a thermal cycler without a heated lid. 19 µl of RT master mix (11 µl of nuclease-free water, 4 µl of 10× RT BUFFER, 2 µl of dNTP mix, and 2 µl of SOLiD™ RT primer) was then prepared on ice. The RT master mix was added to the ligation reaction, pipetted up and down a few times to mix well, and then spun down. After incubating in a thermal cycler with a heated lid at 70°C for 5 min, the RNA sample was immediately snap-cooled on ice. ArrayScript™ Reverse Transcriptase (1 µl) was added to each ligated RNA sample, and the mixture was incubated in a thermal cycler with a heated lid at 42°C for 30 min to perform the reverse transcription reaction. The cDNA was purified using a MinElute^®^ PCR Purification Kit (Qiagen), and then eluted in 10 µl of Buffer EB.

Next, cDNA strands of 60–80 nt (i.e., strands consisting of 10–30 nt miRNA plus ~50 nt of Adapter and terminal sequences) were size-selected using Novex^®^ 10% TBE-Urea gel electrophoresis as described in the Novex^®^ Pre-Cast Gel Electrophoresis Guide (Invitrogen). After cutting the gel vertically into four pieces, the two pieces from the middle of the lane were amplified using in-gel PCR reactions. These reactions were performed by adding 98 µl of PCR mix (76.8 µl of nuclease-free water, 10 µl of 10× PCR Buffer, 8 µl of dNTP Mix, 2 µl of SOLiD™ 5′ PCR Primer, and 1.2 µl of AmpliTaq^®^ DNA Polymerase) and 2 µl of SOLiD 3′ PCR Primer to each gel slice in a 0.2-ml PCR tube. The thermal cycling program was as follows: 95°C for 5 min; 15 cycles of 95°C for 30 s, 62°C for 30 s, and 72°C for 30 s; and a final extension at 72°C for 7 min. The amplified cDNA products were then purified using a PureLink™ PCR Micro Kit (Invitrogen). Templated beads were prepared from this library according to the manufacturer’s instructions and submitted for sequencing on an ABI SOLiD4 machine.

### NGS Data Analysis

Using Galaxy software (30), the output from the ABI SOLiD 4 machine (in csfasta and qual files) was converted to fastq format while removing reads containing color base qualities with a value below 5. Fastq in color space was then converted to nucleotide space using fastq groomer in Galaxy. The adaptor sequence (CGCCTTGGCCGTACAGCAG) was then removed using cutadapt (31), with maximum error rate set to 0.1 and minimum overlap length set to 6. Reads with a selected length of 17–25 bp were converted to DSAP (32) input file format and uploaded to the DSAP web server. Known non-coding RNAs (e.g., tRNA and rRNA) were removed using the Rfam database,^1^ and expression profiles of known miRNAs were reported as Reads Per Kilobase per Million mapped reads (RPKM) by Deep Sequencing Small RNA Analysis Pipeline (DSAP,^2^).

### Northern Blot analysis

Twenty micrograms of total RNA isolated from shrimp stomach was heated at 100°C for 5 min for denaturation, and then put on ice immediately for at least 1 min. The denatured RNA was loaded onto a 14% denaturing polyacrylamide gel with a first run at 50 V for 60 min, and then the voltage was raised to 150 V for ~150 min until the bromophenol blue reached the bottom of the gel. The RNA was then transferred from the gel to a membrane (Hybond XL, GE Healthcare Life Sciences) at 80 V (~340 mA) for 30 min using a wet-transfer system (Hoefer) with 0.5× TBE transfer buffer. After transfer, UV cross-linking was used to fix the RNA to the membrane with 1,200 µJ of energy. Prior to hybridization with the radio-labeled DNA probe, the membrane was kept at 4°C.

The radio-labeled DNA probe was prepared by mixing 1 µl of 25 µM antisense DNA probe with 6 µl of nuclease-free H_2_O and 1 µl of 10× PNK buffer (New England Biolabs), then heating at 100°C for 2 min, and placing on ice immediately. The probe was labeled by adding 1 µl of T4 polynucleotide kinase (10 U/μl; New England Biolabs) and 1 µl of ^32^Pγ-ATP (7,000 Ci/nmole), and then incubating the mixture at 37°C for 2 h. Finally, the mixture was heated at 100°C for 5 min, and passed through a mini Quick Spin™ Oligo Columns (Qiagen) to remove any remaining free isotope. The ^32^P-labeled DNA probe was stored at −20°C for later use.

For the hybridization step, the membrane was first pre-hybridized in hybridization buffer (0.5 M NaH_2_PO_4_, 7% SDS, 1 mM EDTA, pH 7.2) at 37°C for 30 min. Hybridization was then performed in 5 ml of hybridization buffer with 10 µl of radio-labeled DNA probe at 37°C overnight. After hybridization, the membrane was washed three to four times with 2× SSC at 37°C for 15 min and then exposed to Kodak BioMax MR film with an intensifying screen for several days at −80°C.

### Real-time PCR Quantification of Mature miRNA

Total RNA isolated from shrimp stomach was treated by on-column DNase digestion (RNase-free DNase set; Qiagen) according to the manufacturer’s instructions. Reverse transcription was performed using the miScript II RT kit (Qiagen) according to the manufacturer’s instructions. Briefly, DNase-treated RNA was reverse transcribed into cDNA using the 5× miScript HiSpec buffer for quantification of mature miRNAs. miRNA expression was analyzed using the miScript SYBR Green PCR kit (Qiagen) on a CFX96 Real-Time Detection System (Bio-Rad) according to the manufacturer’s instructions. Each reaction consisted of 2.5 µl of diluted cDNA was mixed with 12.5 µl of 2× QuantiTect SYBR Green PCR Master Mix, 2.5 µl of 10× miScript Universal Primer, and 2.5 µl of 10× miScript Primer (specific for miR-10a or the U6 internal control, Qiagen).

### *In Vivo* Experiments

Chemically synthesized microRNA inhibitors (anti-miR-10a and anti-miRNA control) with proprietary stabilizing modifications were purchased from Dharmacon. For *in vivo* experiments, experimental groups of 20 shrimps were injected with either the anti-miR-10a (sense strand: 5′-UACCCUGUAGAUCCGAAUUUGU-3′; 0.5 nmol/shrimp) and WSSV (10,000× dilution of the virus stock) in 50 µl of PBS or with the negative control anti-miRNA (sense strand: 5′-ACAACCUCCUAGAAAGAGUAGAUU-3′; 0.5 nmol/shrimp) and WSSV (10,000× dilution of the virus stock) in 50 µl of PBS; or with 50 µl of PBS and WSSV (10,000× dilution of the virus stock) only (positive control). Pleopod and stomach samples were collected from four randomly selected shrimp in each group at 24 and 48 hpi.

### Determination of WSSV Copy Number

To determine WSSV copy number, a commercial WSSV diagnostic kit (IQ REAL™ WSSV Quantitative System, GeneReach Biotechnology Corp.) was used according to the manufacturer’s instructions and as described previously (33). Briefly, genomic DNA extracted from the shrimp pleopod samples was isolated using the silica-extraction procedure, and then subjected to real-time PCR analysis. The results were calculated and WSSV copy number were determined according to the manufacturer’s instructions. Data are presented as the mean ± SD from the four individual shrimp. Data were analyzed using the Student’s *t*-test. *p* < 0.05 was considered statistically significant.

### Western Blotting

Total protein was extracted from shrimp stomach by a hypotonic PBS with a protease inhibitor cocktail tablet (Roche). Extracted samples (15 μg) were separated on 15% SDS-PAGE and transfer to a PVDF membrane. WSSV IE1 was detected with a rabbit anti-WSSV IE1 (34) antibody and a goat anti-rabbit secondary antibody (GeneTex). WSSV ICP11 and VP28 were, respectively, detected with rabbit anti-WSSV ICP11 and anti-VP28 antibodies (35, 36) and the same goat anti-rabbit secondary antibody (GeneTex). Shrimp actin which was used as the internal control, was detected with rabbit anti-actin antibody (GeneTex) and also used the same goat anti-rabbit secondary antibody (GeneTex). Target signals were visualized using ECL western blotting detection reagents (GE Healthcare) and ImageQuant™ LAS 4000 digital imaging system (GE Healthcare) according to the manufacturer’s instructions.

### Plasmid Construction and Dual-Luciferase Assays

All of the primers used for plasmid construction are listed in Table 1. The parental plasmid used for the dual-luciferase assay was constructed as described previously (37). Basically, the T7 promoter of plasmid pGL3 (Promega) was replaced by the WSSV *ie1* promoter (positions −94 to +52) (17), and the *ie1*/pGL3 plasmid was inserted with the appropriate viral 5′-UTR sequence to obtain *ie1*/pGL3/*wssv* 5′-UTR firefly luciferase expression constructs. The mutation constructs were produced by rolling circle PCR using *ie1*/pGL3/*wssv* 5′-UTR as templates.

**Table 1:** Primers used in the dual-luciferase assays in this study.

Primer name	Sequence^a^
wssv234 5′ untranslated region (UTR)-SmaI-F	5′-aaacccgggCATTCTTCACACCATAAAAGGACA-3′
wssv234 5′ UTR-NcoI-R	5′-aaaccatggCTTGACGGTTTGTTTCTGTCTAC-3′
vp26 5′ UTR-SmaI-F	5′-aaacccgggACTTCATCCGGTTCGATGTAG-3′
vp26 5′ UTR-NcoI-R	5′-aaaccatggTTTTCTTTGTTTTAGATGGAAGTTC-3′
vp28 5′ UTR-SmaI-F	5′-aaacccgggGTCCTGTTACGTACTCTGTGGTTT-3′
vp28 5′ UTR-NcoI-R	5′-aaaccatggGACGAGTTTTTTTCTTTATCGAACG-3′
wssv102 5′ UTR-SmaI-F	5′-aaacccgggTGAAATAGAAGACGTTCAAGTACAG-3′
wssv102 5′ UTR-NcoI-R	5′-aaaccatggCGCTATCTATTAAATCCAATAATTG-3′
vp26 5′-mut-F	5′-TGTCAAGAACTAACTAGCTGGATCCAAC-3′
vp26 5′-mut-R	5′-AGCACCATATACCCAGAAAGG-3′
vp28 5′-mut-F	5′-TAGATAATAACCAAGCAACGTTCGATAAAGAA-3′
vp28 5′-mut-R	5′-GTCGTTTTGTCGGCGAGGAC-3′
wssv102 5′ UTR-mut-F	5′-CCCTGGAGAAGAAGAGGAACTTCCTTCA-3′
wssv102 5′ UTR-mut-R	5′-TGTTGATGTTGTTCTATTTCAGGGATATCG-3′

*^a^Lower case nucleotides indicate enzyme cutting sites; underlining indicates mutated nucleotides*.

For transfection, synthesized miR-10a mimic and two LNA™ microRNA inhibitors (anti-miR-10a and anti-miRNA control) were purchased from Exiqon. Sf9 insect cells were seeded into a 24-well plate (2 × 10^5^ cells/well) and grown overnight. Plasmids (internal control *Renilla* luciferase, *ie1*/pGL3/*wssv* 5′-UTR firefly luciferase and miR-10a mimic) were transfected together with miR-10a mimic, anti-miR-10a, or anti-miRNA control inhibitor using a Cellfectin transfection kit (Thermo) according to the manufacturer’s protocol. Cells were harvested 2 days after transfection, and a dual-luciferase assay system (Promega) was used to analyze luciferase activities according to the manufacturer’s protocol. Transfection assays were performed in triplicate with three independent experiments. Data are presented as the mean ± SD from the three independent triplicate experiments. Data were analyzed using the Student’s *t*-test. *p* < 0.05 was considered statistically significant.

## Results

### Global Analysis of microRNA Expression Patterns after WSSV Infection in Shrimp

To reveal which host miRNAs might be involved in host–virus interactions, the SOLiD4 NGS platform was used to perform a time course analysis of miRNA expression at 0, 12, 24, 36, and 48 h postinfection (hpi) in shrimp stomach. Subsequent bioinformatic analysis identified 249 host miRNAs that matched known miRNAs in the miRBase database (version 17), while 78 viral miRNA candidates were found using mireap and miRdeep2. The miRNA expression values were normalized by RPKM, and DSAP analysis was used to produce differential expression profiles of the host miRNAs. Table 2 lists miRNAs that were no longer detected after WSSV infection, and also shows four of the most dramatically upregulated miRNAs: miR-10a, miR-252a, miR-252b, and miR-263b. In the present study, we focus on the most highly upregulated host miRNA, miR-10a. We note in passing that this particular miRNA is also upregulated in several types of human cancer cell (38–40).

**Table 2:** Differential expression of host microRNAs (miRNAs) in white spot syndrome virus-infected shrimp.

Name	Sequence	Homology^a^	RPKM				
			0 hpi	12 hpi	24 hpi	36 hpi	48 hpi
**(A) miRNAs that were upregulated after infection**							
miR-10a	5′-UACCCUGUAGAUCCGAAUUUGU-3′	ssc-miR-10a	0	575.8	501.3	319.1	226.3
miR-252a	5′-CUAAGUACUAGUGCCGCAGGAGU-3′	sko-miR-252a	0	93.7	11.3	70.0	33.3
miR-252b	5′-CUAAGUAGUAGUGCCGCAGGUAA-3′	sko-miR-252b	0	73.6	208.4	62.3	26.6
miR-263b	5′-CUUGGCACUGGAAGAAUUCAC-3′	nlo-miR-263b	0	60.3	95.8	7.8	33.3
**(B) miRNAs that were no longer detected after infection**							
miR-397	5′-UCAUUGAGUGCAGCGUUGAUG-3′	cis-miR-397	496.9	0	0	0	0
miR-171b	5′-UUGAGCCGUGCCAAUAUCACG-3′	tae-miR-171b	196.4	0	0	0	0
miR-590-3p	5′-UAAUUUUAUGUAUAAGCUAGU-3′	ppy-miR-590-3p	34.7	0	0	0	0
miR-263b	5′-CUUGGCACUGGGAGAAUUCAC-3′	aga-miR-263b	23.1	0	0	0	0
bantam	5′-UGAGAUCAUUGUGAAAGCUAAUU-3′	api-bantam	23.1	0	0	0	0
miR-281*	5′-UGUCAUGGAAUUGCUCUCUUU-3′	cqu-miR-281*	11.6	0	0	0	0
miR-281-1*	5′-AAGAGAGCUGUCCGUCGACAGU-3′	dya-miR-281-1*	11.6	0	0	0	0
miR-71	5′-UGAAAGACAAGGGUAGUGAGAUG-3′	lgi-miR-71	11.6	0	0	0	0
miR-396c	5′-UUCCACGGCUUUCUUGAACUU-3′	pab-miR-396c	11.6	0	0	0	0

*^a^Although miRBase version 17 was used to search matches with known miRNAs, the homology names in this table are from miRBase version 20*.

*RPKM, reads per kilobase per million mapped reads; hpi, hours postinfection*.

### Additional Evidence That the microRNA miR-10a Was Upregulated after WSSV Infection

**Figure 1:**
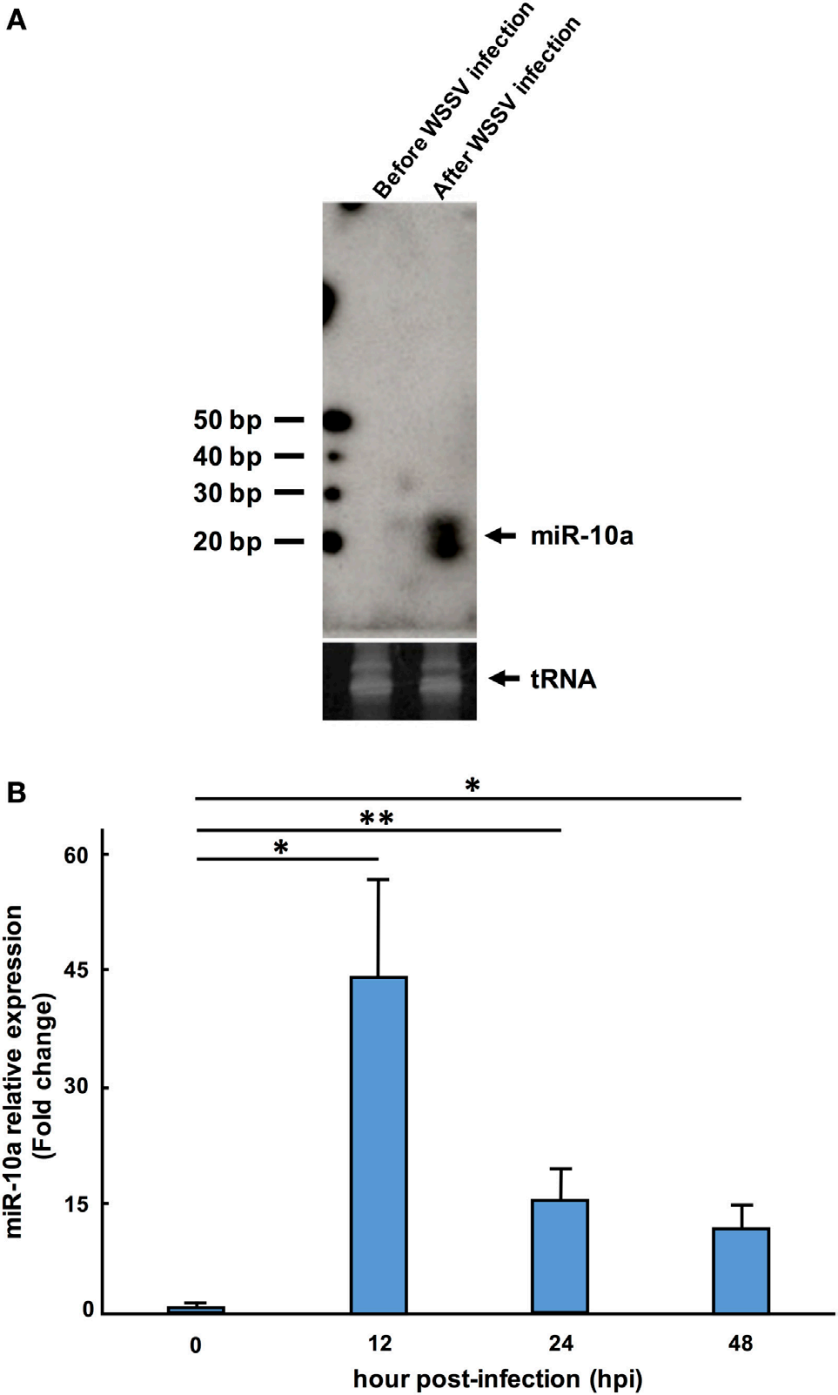
Expression level of miR-10a after white spot syndrome virus (WSSV) infection. **(A)** Northern blot of shrimp miR-10a. Total RNA extracted from shrimp stomach before WSSV infection (0 dpi) and after WSSV infection (48 dpi) was blotted with γ[^32^P]-labeled miR-10a probe (upper panel). tRNA was used as a loading control (lower panel). **(B)** Real-time PCR analysis of the expression of miR-10a at 0, 12, 24, and 48 hpi in shrimp stomach. 2^−ΔΔCt^ was used to analyze the expression of miR-10a relative to the expression of shrimp U6 (internal control). Data are presented as mean ± SD from three shrimp at each time point (*p < 0.05 and **p < 0.01 by Student’s *t*-test).

In confirmation of the NGS data, a Northern blotting analysis showed that the miR-10a signal was negligible before WSSV infection and significantly upregulated after WSSV infection (Figure 1A). The presence of two miR-10a bands with a size difference of ~1 nt suggests that two miR-10a isoforms are induced after WSSV infection, and from our raw NGS data, we infer that the second 21-nt isoform is produced by a 1-nt deletion at the 5′ end of the 22-nt miR-10a. Real-time PCR further showed that the expression level of mature miR-10a was significantly elevated after WSSV infection (Figure 1B). At 12 hpi (i.e., about halfway through WSSV’s 24 h replication cycle), expression was almost 40 times higher than at 0 hpi (Figure 1B). Both of these results are consistent with the NGS data in Table 2.

### miR-10a Inhibitor Reduces WSSV Replication *In Vivo*

**Figure 2:**
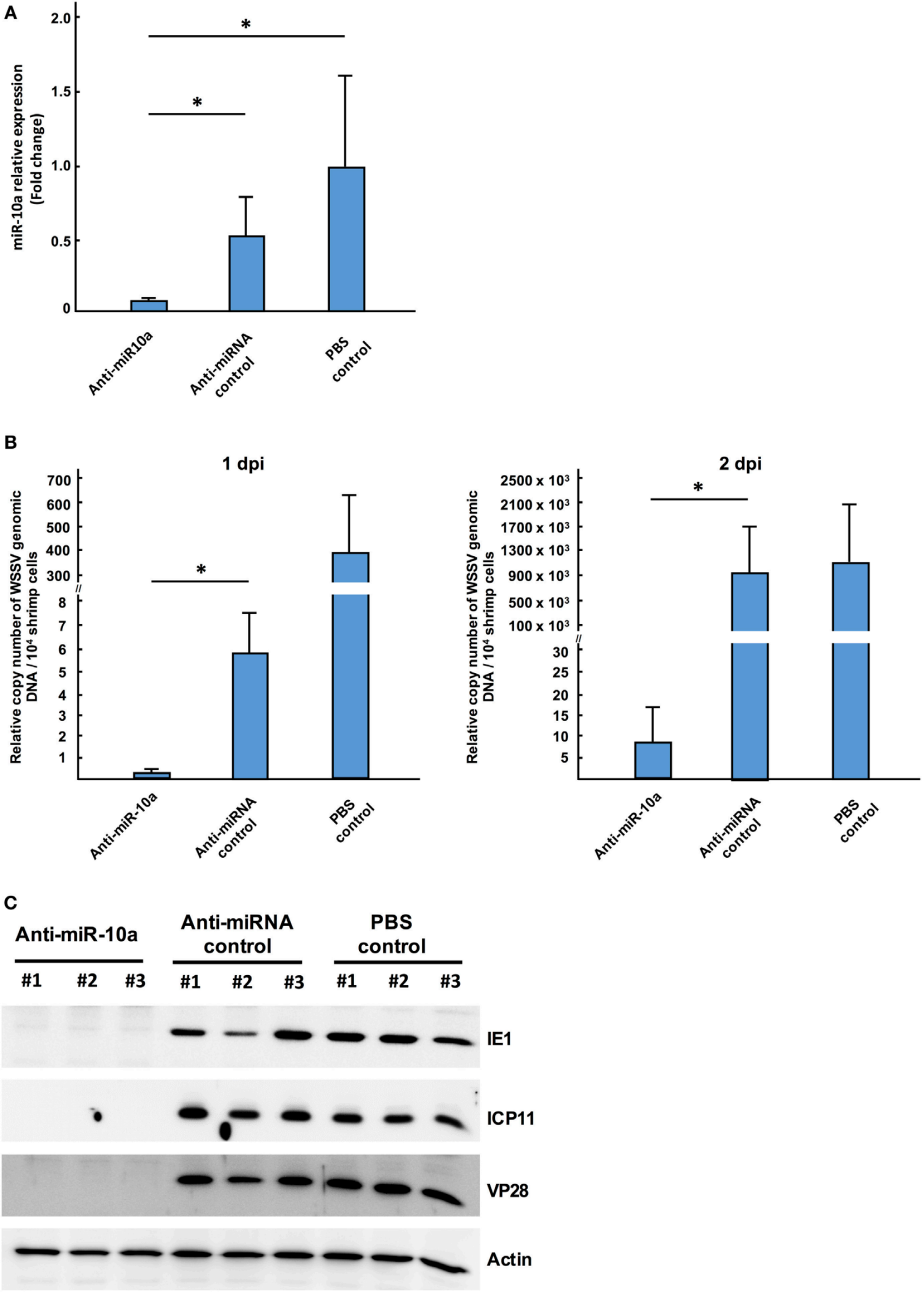
The role of host miR-10a in white spot syndrome virus (WSSV)-infected shrimp. Shrimp (*L. vannamei*; mean weight 7 g) were injected simultaneously with WSSV and control anti-miRNA inhibitor (1 nmol/shrimp); WSSV and anti-miR-10a inhibitor (1 nmol/shrimp); WSSV and phosphate-buffered saline (PBS). **(A)** Real-time PCR analysis of miR-10a expression 1 day after the injection of anti-miR-10a inhibitor. miR-10a expression levels were determined in cDNA derived from shrimp pleopods. 2^−ΔΔCt^ was used to analyze the expression of miR-10a, and results are presented relative to the expression of shrimp U6. Values shown are the mean ± SD from four shrimp at each time point (*p < 0.05 by Student’s *t*-test). **(B)** An IQ REAL™ WSSV Quantitative System kit was used to determine the WSSV copy number in shrimp pleopods. Values shown are the mean ± SD from four shrimp at each time point (*p < 0.05 by Student’s *t*-test.) **(C)** Specific anti-IE1, anti-ICP11 and anti-VP28 antibodies were used to detect WSSV protein levels in shrimp stomach of anti-miR-10a inhibitor treated shrimp, non-specific microRNA inhibitor treated shrimp, and PBS-injected shrimp. Shrimp actin was detected with anti-actin antibody as internal control.

To investigate whether or not miR-10a plays a functional role during WSSV infection, shrimp were injected with a mixture of miR-10a inhibitor and WSSV, and the WSSV infection status was monitored using an IQ REAL™ WSSV Quantitative System (GeneReach Biotechnology Corp.). First, to confirm the specificity of the miR-10a inhibitor, we used quantitative RT-PCR to analyze the expression level of miR-10a in the pleopods of challenged shrimp at 1 dpi. As shown in Figure 2A, in four randomly selected shrimp from each group, miR-10a expression was significantly decreased in the miR-10a inhibitor treated shrimp but not in the non-specific anti-microRNA inhibitor or the PBS-injected shrimp. In shrimp treated with the miR-10a inhibitor, the WSSV copy number was significantly decreased at 1 and 2 dpi (Figure 2B). Finally, western blots (Figure 2C) show that the WSSV proteins IE1, ICP11, and VP28 were all significantly downregulated in shrimp treated with the miR-10a inhibitor.

Taken together, these results show that inhibition of miR-10a with the miR-10a inhibitor reduced WSSV replication. This in turn suggests that host miR-10a plays an important, positive role in WSSV replication.

### Shrimp miR-10a Interacts with the 5′ UTR of Viral Genes

**Figure 3:**
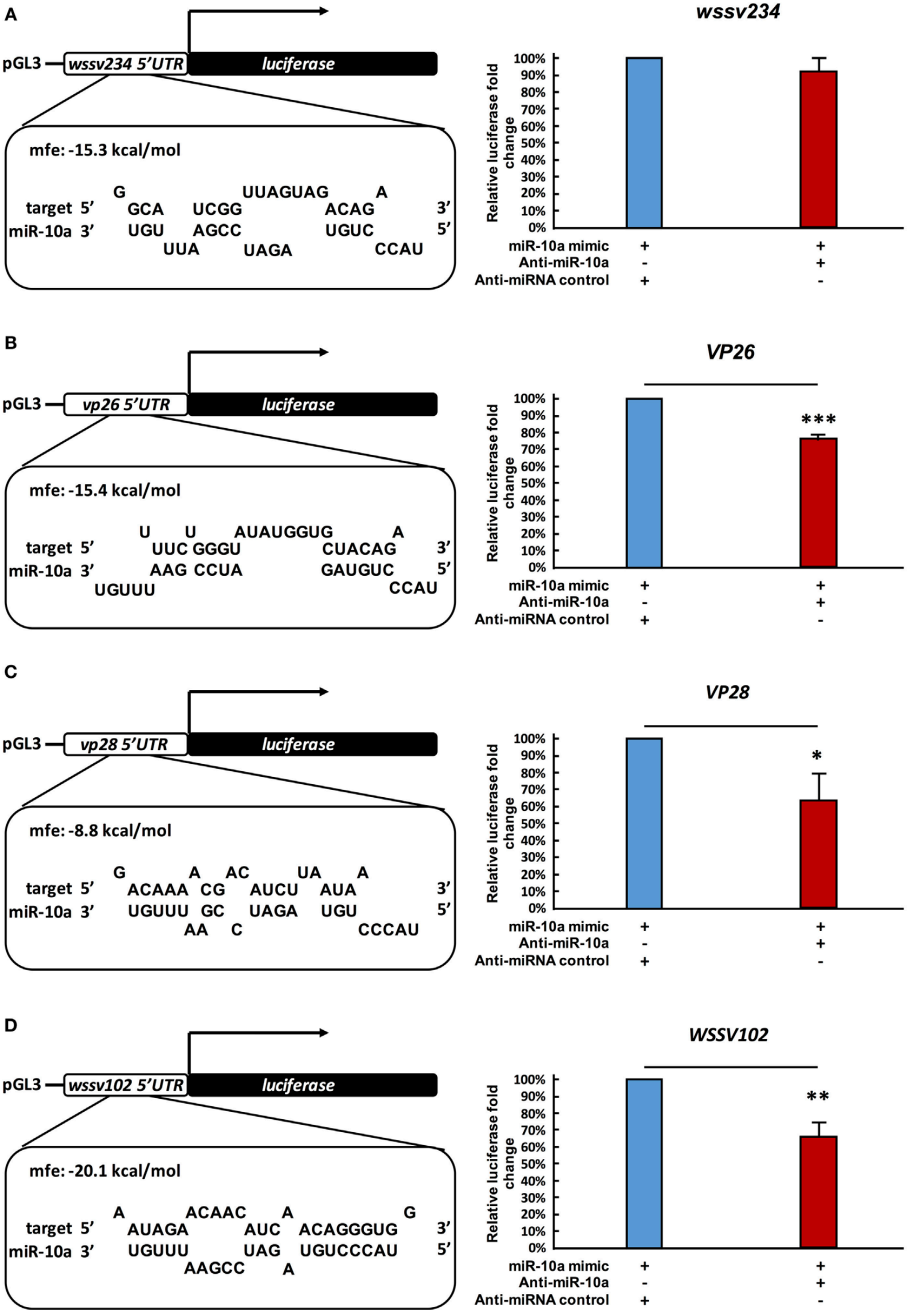
Suppression of miR-10a significantly reduces the expression of at least three WSSV genes. **(A–D)** Luciferase reporter assay for *wssv234, vp26, vp28*, and *wssv102*, respectively. Sf9 cells were transfected as indicated with the respective *ie1*/pGL3/5′-UTR firefly luciferase expression construct (500 ng) plus miR-10a mimic (32 pmol), along with either anti-miR-10a (32 pmol) or the anti-miRNA control (32 pmol). At 48 h post-transfection, firefly luciferase signals were normalized relative to those of the *Renilla* luciferase internal control, and data are shown relative to the anti-miRNA control (set to 100%). Data represent mean ± SD from three independent experiments; *p < 0.05, **p < 0.01, and ***p < 0.005 by Student’s *t*-test.

A previous study reported that in a mouse cell line, miR-10a enhanced the translation of ribosomal genes by binding to their 5′ UTR (41). Based on our above results, we now further hypothesize that in a similar way, shrimp miR-10a might likewise be enhancing the expression of some WSSV genes by interacting with their 5′ UTR. Using alignment software Vector NTI, we, therefore, conducted a search on the 5′ UTR of WSSV genes to look for sequences that might interact with miR-10a, and the “RNA hybrid” program^3^ was then used to calculate the minimum free energy (mfe) of these possible interactions. Sequences that were predicted to hybridize with a minimum free energy of <−5 kcal/mol are list in Table S1 in Supplementary Material. (Although two isoforms were detected in Figure 1A, we note that the 22-nt miR-10a and the truncated 21-nt miR-10a are both predicted to target the same genes.) From these results, we selected four candidate genes at random for further testing: one immediate early gene *wssv234* (Figure 3A left panel), two major structural protein genes *vp26, vp28* (Figures 3B,C left panel), and one gene *wssv102* (Figure 3D left panel) with unknown function. We then used miR-10a inhibitor and a luciferase assay to investigate the effect of miR-10a on plasmids constructed from the 5′ UTR of these four WSSV candidate genes in Sf9 cells. As shown in Figure 3, we found that miR-10a inhibitor significantly reduced the luciferase signals controlled by the 5′ UTR of *vp26, vp28*, and *wssv102*. The reductions in luciferase activity were approximately 20–40% compared to the negative control anti-miRNA inhibitor (Figures 3B–D right panel). Only the 5′ UTR of *wssv234* failed to show any significant difference in luciferase activity between the miR-10a inhibitor and the negative control anti-miRNA inhibitor (Figure 3A, right panel).

**Figure 4:**
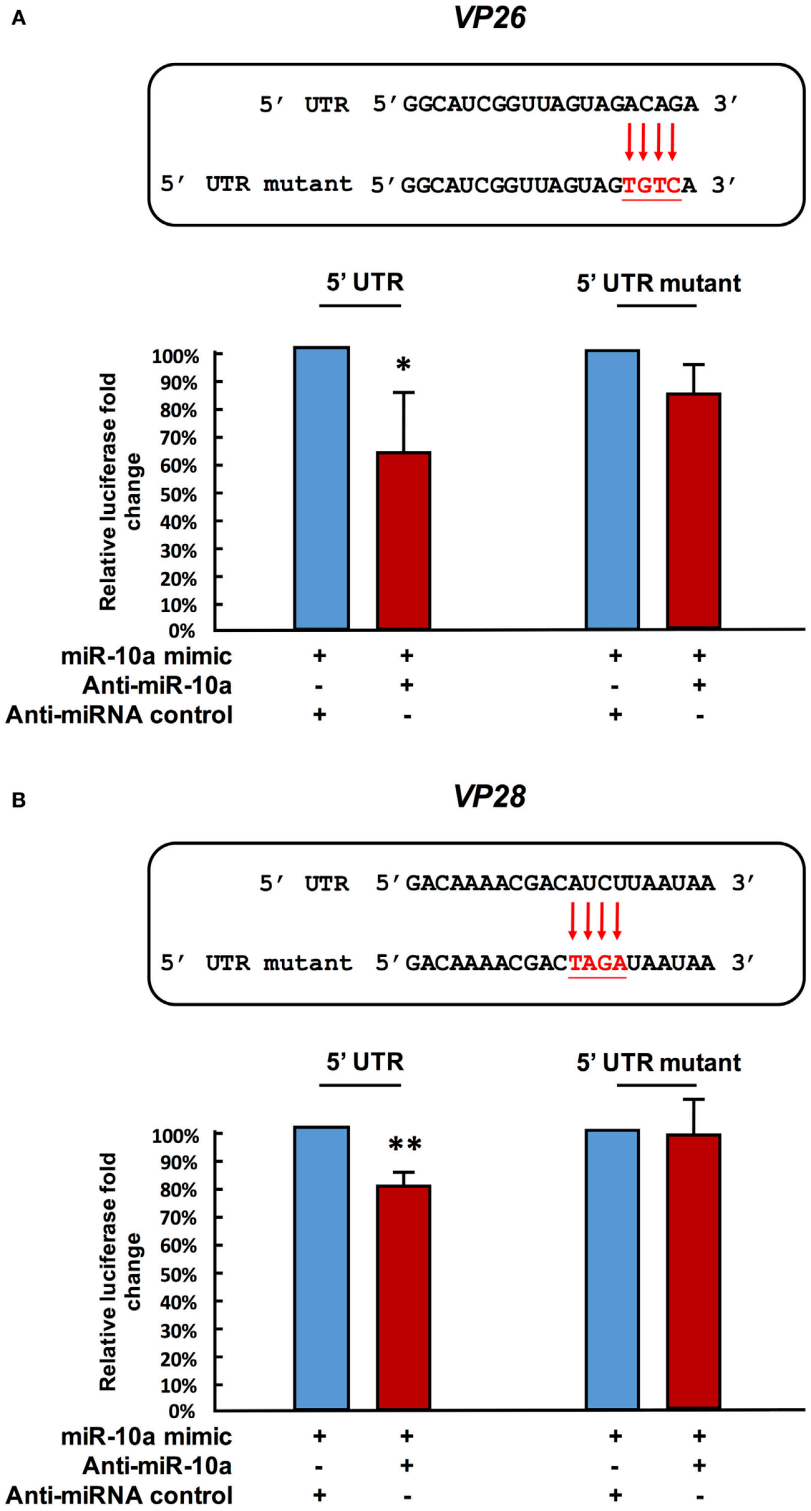

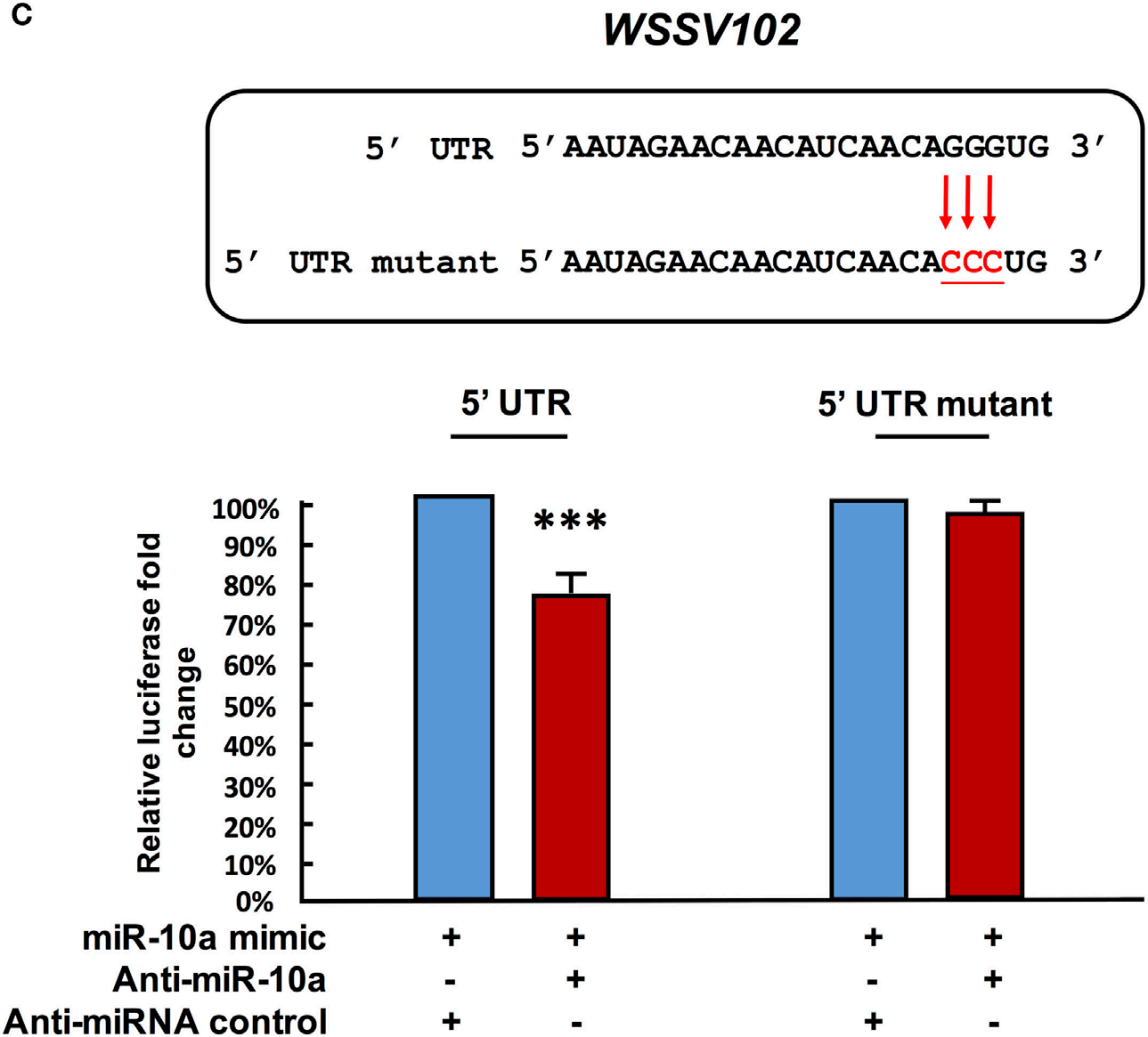
Mutation of the target gene’s 5′ UTR prevents the downregulation caused by miR-10a suppression. Sf9 cells were transfected with the miR-10a minic (23 pmole) plus 500 ng of the non-mutant or mutant ie1/pGL3/5′-UTR firefly luciferase expression construct for **(A)** VP26, **(B)** VP28, and **(C)** WSSV102, along with either anti-miR-10a (32 pmol) or the anti-miRNA control (32 pmol) as indicated. At 48 h post-transfection, the firefly luciferase signals were normalized relative to those of the *Renilla* luciferase internal control, and data are shown relative to the anti-miRNA control (set to 100%). Data represent mean ± SD from three independent experiments; *p < 0.05, **p < 0.01, and ***p < 0.005 by Student’s *t*-test. The mutated nucleotides are shown in bold.

Next, in order to further verify the interaction between miR-10a and the 5′ UTR of the viral genes, we abolished some of the predicted base-pairing target sites of miR-10a by constructing 5′ UTR mutant plasmids for *vp26, vp28*, and *wssv102* (Figures 4A–C, upper panel). We then used the same luciferase assay to compare activities in the SF9 cell line. Since the miR-10a inhibitor failed to significantly reduce the firefly luciferase signal of any of these mutants (Figures 4A–C, lower panel), we concluded that shrimp miR-10a upregulated the expression of *vp26, vp28*, and *wssv102* by targeting the 5′ UTR of these viral genes.

## Discussion

As noted above, although most miRNAs target the 3′ UTR and act as suppressive factors for gene regulation, miRNAs have also been reported to upregulate their targets (41–45). In the present study, we now show that the shrimp microRNA miR-10a enhances the expression of WSSV structural and non-structural proteins by targeting the 5′ UTR of the viral mRNAs. The biological importance of this enhancement was further shown by the results of our *in vivo* challenge experiment, where WSSV infection was reduced when miR-10a was specifically inhibited (Figures 2B,C).

After alignment with Vector NTI, preliminary predictions by RNAhybrid suggested that miR-10a might target a number of important structural and non-structural WSSV genes, including *vp24, vp19, icp35, DNA polymerase, ie1, icp11*, and *rr2* (Table S1 in Supplementary Material). We note, however, that, like *wssv234, vp26, vp28*, and *wssv102*, these candidate targets will need to be tested experimentally because while the number of matched nucleotides, their location in the 5′ UTR of the target mRNA, and the predicted free energy of the interaction all seem to be important, these factors alone do not appear to provide a reliable indication of whether or not such an interaction in fact occurs.

**Figure 5:**
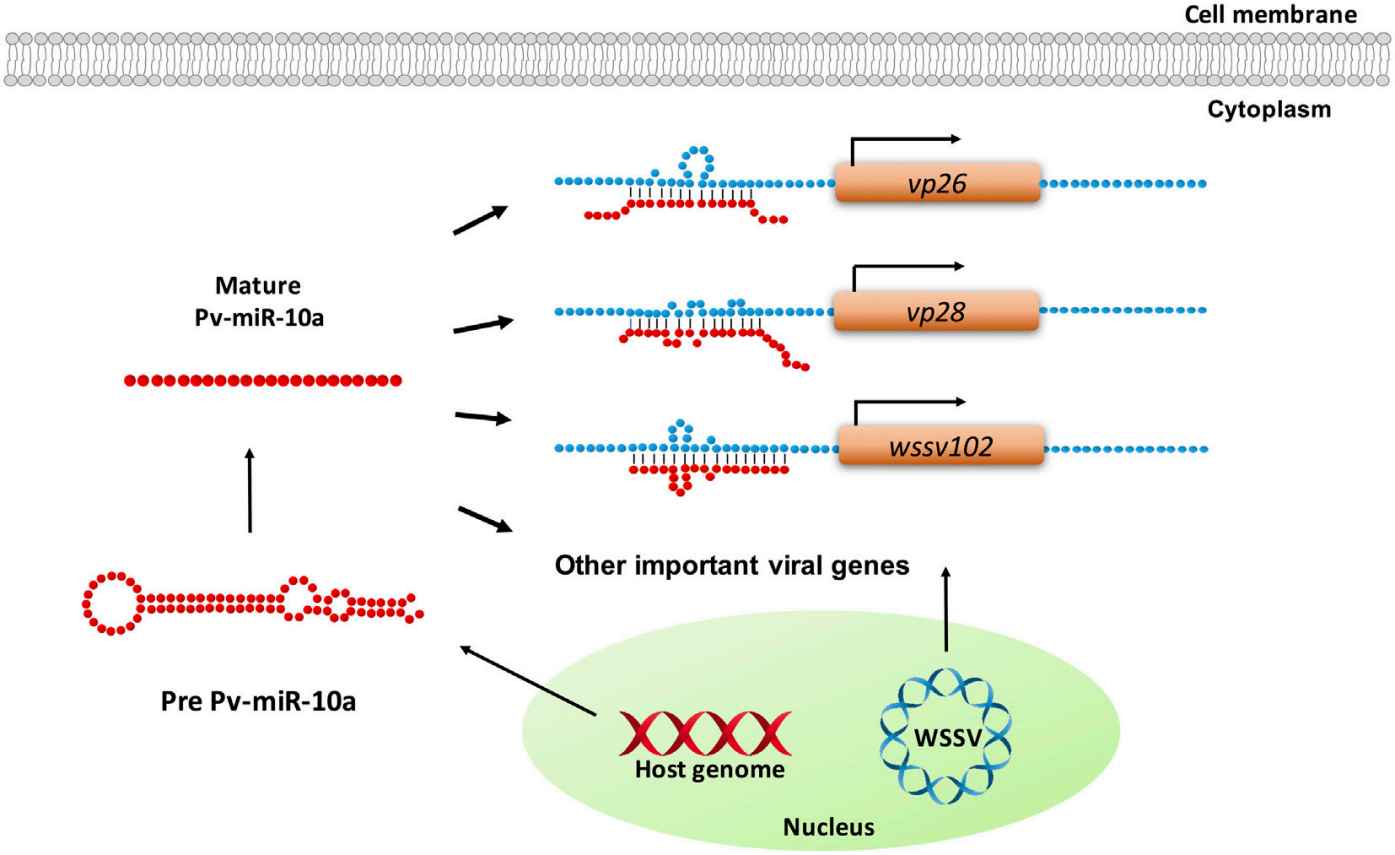
Schematic representation of how shrimp miR-10a is co-opted by white spot syndrome virus (WSSV) to enhance viral replication. Pv-miR-10a upregulates the expression of at least three WSSV genes (*vp26, vp28*, and *wssv102*) by targeting their 5′ untranslated region. Alignment followed by RNAhybrid prediction suggests that several other important WSSV genes are also likely to be upregulated by host miR-10a.

In the present study, the ability of miR-10a to enhance the expression of the candidate viral genes listed in Table S1 in Supplementary Material was experimentally tested in only four genes. While the results for *wssv234* were negative (Figure 3A), miR-10a upregulated the expression of two important structural proteins, VP26 and VP28, and of one viral protein with unknown function, *wssv102* (Figures 3B–D). VP28 is a major envelope protein, while VP26 is a tegument protein (46), and several studies have reported that silencing either of these two genes by RNA interference in shrimp led to reduced mortality and provided protection against WSSV infection (47, 48). These two genes—and the augmentation of their expression by miR-10a—therefore, seem critical for viral replication. Meanwhile, although further experiments will be needed to clarify the role of WSSV102 during WSSV infection, we found that the protein was located in the nucleus (Figure S1 in Supplementary Material), and an NCBI conserved domain search of its amino acid sequence identified an STP6 acidic domain and a Paf1 domain. WSSV102 might, therefore, function as a transcription regulator and interact with RNA polymerase II. Taking these results together, we, therefore, conclude that shrimp miR-10a is being co-opted by these and other WSSV genes in order to enhance WSSV replication. A schematic representation of this proposed model is shown in Figure 5.

## Author Contributions

S-TK and J-YH designed and performed *in vitro*/*in vivo* experiments, analyzed data, and wrote the manuscript. I-TC and L-KC analyzed and discussed data. S-SL and G-HK participated in manuscript preparation and discussed data. C-YC and C-FL conceived the idea, designed the research, discussed data, and supervised this work.

## Conflict of Interest Statement

The authors declare that the research was conducted in the absence of any commercial or financial relationships that could be construed as a potential conflict of interest.

## Footnotes

^1^http://rfam.xfam.org.

^2^http://dsap.cgu.edu.tw/.

^3^http://bibiserv.techfak.uni-bielefeld.de/rnahybrid/.

## Abbreviations

WSSV, white spot syndrome virus; NGS, next-generation sequencing; miRNA, microRNA; UTR, untranslated region; PBS, phosphate-buffered saline; RPKM, Reads Per Kilobase per Million mapped reads.
