# Supplementary material for: Shrimp miR-10a Is Co-opted by White Spot Syndrome Virus to Increase Viral Gene Expression and Viral Replication
Source: Front Immunol. 2017 Sep 6;8:1084. doi: 10.3389/fimmu.2017.01084 (PMC5592198; doi:10.3389/fimmu.2017.01084)
Supplement: Supplementary file 1 [file table_1.docx]

**Supplementary Table 1. Other viral genes which might be targeted by shrimp miR-10a.**

| Viral gene | Predicted miR-10a target region on the 5’UTR of the viral gene^1^ | Mfe (kcal/mol) |
| --- | --- | --- |
| *wssv234* | 5’-GGCAUCGGUUAGUAGACAGA-3’ | -15.3 |
| *vp26* | 5’-UUUCUGGGUAUAUGGUGCUACAGA-3’ | -15.4 |
| *vp28* | 5’-GACAAAACGACAUCUUAAUAA-3’ | -8.8 |
| *wssv102* | 5’-AAUAGAACAACAUCAACAGGGUGG-3’ | -20.1 |
| *vp24* | 5’-UUUCGAGUAUGCUGA-3’ | -10.3 |
|  | 5’-ACGGCAGUUGCUGCU-3’ | -11.7 |
| *vp19* | 5’-AGCGGACGCCGUGGAUCUA-3’ | -16.8 |
|  | 5’-CUUCAUCAAACAGA-3’ | -9.3 |
| *ICP35* | 5’-CACGAGUGUAUAUAUAGGA-3’ | -15.2 |
| *DNApol* | 5’-CUCGGAACCCACAGCUCGG-3’ | -14.5 |
| *ie1* | 5’-CGAUUCAGUCACAA-3’ | -10.9 |
| *rr2* | 5’-UAUAAAUAUGGCCACUUCUCAC-3’ | -11.1 |
| *icp11* | 5’-UUCUUGAAGAGGA-3’ | -10.5 |
|  | 5’-AUGGCCACCUUCCAG-3’ | -7.7 |
| *wssv400* | 5’-GAUGAAGAGGACAAAAACACAGGGUA-3’ | -20.8 |
| *wssv348* | 5’-GACGAAAUAUCACAGGGUGU-3’ | -20.3 |

1: Underscoring indicates nucleotides that are predicted to match.
