# Supplementary material for: Shrimp miR-10a Is Co-opted by White Spot Syndrome Virus to Increase Viral Gene Expression and Viral Replication
Source: Front Immunol. 2017 Sep 6;8:1084. doi: 10.3389/fimmu.2017.01084 (PMC5592198; doi:10.3389/fimmu.2017.01084)
Supplement: Supplementary file 2 [file image_1.pdf]

Supplementary Figure

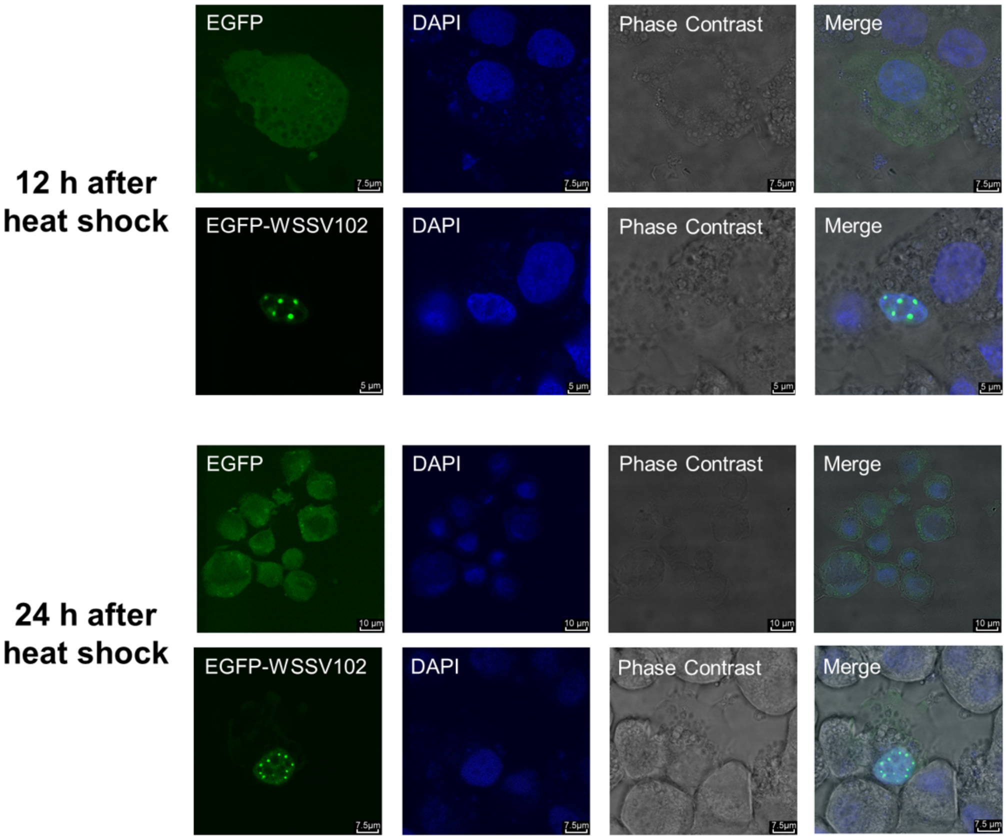

Supplementary figure 1. Cellular localization of WSSV102. *wssv102* was inserted into the EGFP expression plasmid pIZ/EGFP with the primer sets (*wssv102*-BamHI-F, 5'-AAAggatccATGGAAGAAACAATAACCCT-3'; and *wssv102*-EcoRV-R, 5'-AAAgatatcTAGGAAGAAACGTCCAT-3'). The resulting construct, pIZ/EGFP/*wssv102*, or the parent plasmid control (pIZ/EGFP), were transfected into Sf9 cells by Cellfectin (Thermo) according to the manufacturer's protocol. At 12h and 24 h after heat shock, the location of the expressed EGFP or EGFP-WSSV102 fusion protein was observed in the transfected cells using a confocal fluorescence microscope. The cell nuclei were stained with DAPI.
